# The MLL–Menin Interaction is a Therapeutic Vulnerability in *NUP98*-rearranged AML

**DOI:** 10.1097/HS9.0000000000000935

**Published:** 2023-07-27

**Authors:** Milad Rasouli, Helen Blair, Selina Troester, Katarzyna Szoltysek, Rachel Cameron, Minoo Ashtiani, Anja Krippner-Heidenreich, Florian Grebien, Gerard McGeehan, C. Michel Zwaan, Olaf Heidenreich

**Affiliations:** 1 Princess Maxima Center for pediatric Oncology, Utrecht, The Netherlands; 2 Department of Pediatric Hematology/Oncology, Erasmus MC-Sophia Children’s Hospital, Rotterdam, The Netherlands; 3 Wolfson Childhood Cancer Research Centre, Translational and Clinical Research Institute, Newcastle University, Newcastle upon Tyne, United Kingdom; 4 Institute for Medical Biochemistry, University of Veterinary Medicine Vienna, Austria; 5 Maria Sklodowska-Curie Institute – Oncology Center, Gliwice Branch, Poland; 6 Syndax Pharmaceuticals, Waltham, MA, USA

## Abstract

Chromosomal translocations involving the *NUP98* locus are among the most prevalent rearrangements in pediatric acute myeloid leukemia (AML). AML with *NUP98* fusions is characterized by high expression of *HOXA* and *MEIS1* genes and is associated with poor clinical outcome. NUP98 fusion proteins are recruited to their target genes by the mixed lineage leukemia (MLL) complex, which involves a direct interaction between MLL and Menin. Here, we show that therapeutic targeting of the Menin–MLL interaction inhibits the propagation of *NUP98*-rearrranged AML both ex vivo and in vivo. Treatment of primary AML cells with the Menin inhibitor revumenib (SNDX-5613) impairs proliferation and clonogenicity ex vivo in long-term coculture and drives myeloid differentiation. These phenotypic effects are associated with global gene expression changes in primary AML samples that involve the downregulation of many critical NUP98 fusion protein-target genes, such as *MEIS1* and *CDK6*. In addition, Menin inhibition reduces the expression of both wild-type *FLT3* and mutated *FLT3*-ITD, and in combination with FLT3 inhibitor, suppresses patient-derived *NUP98*-r AML cells in a synergistic manner. Revumenib treatment blocks leukemic engraftment and prevents leukemia-associated death of immunodeficient mice transplanted with NUP98::NSD1 FLT3-ITD-positive patient-derived AML cells. These results demonstrate that *NUP98*-rearranged AMLs are highly susceptible to inhibition of the MLL–Menin interaction and suggest the inclusion of AML patients harboring *NUP98* fusions into the clinical evaluation of Menin inhibitors.

## Graphical abstract

**Figure:**
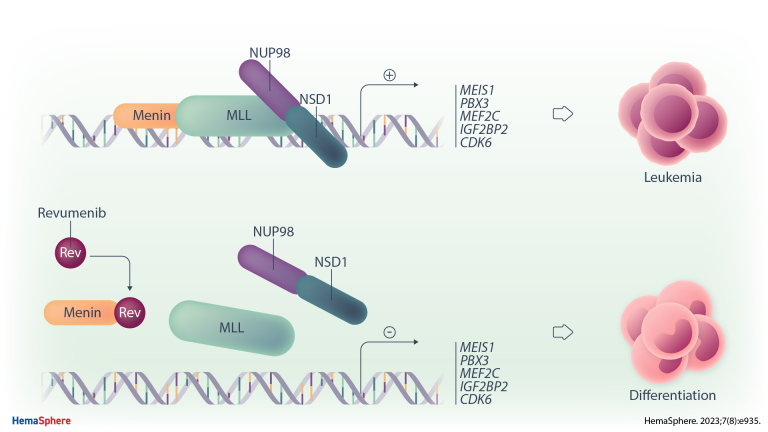

## INTRODUCTION

More than 50% of pediatric and adolescent acute myeloid leukemia (AML) cases are characterized by chromosomal rearrangements that give rise to leukemic fusion genes.^1^ Translocations of the *Nucleoporin 98 (NUP98*) gene are among the most common recurrent translocations in pediatric AML, representing 4% of all fusion events. Internal tandem duplications in the *Fms-like tyrosine kinase 3 gene* (*FLT3*-ITD) are a common genetic alteration in *NUP98*-rearranged (*NUP98*-r) AML (>70%),^2^ which has been associated with a dismal clinical outcome.^3^

The wild-type NUP98 protein contains 2 N-terminal intrinsically disordered regions featuring 38 FG/GLFG repeats, which are separated by a GLEBS motif. This intrinsically disordered FG-rich region is involved in trafficking of RNA molecules through the nuclear membrane and participates in the formation of biomolecular condensates.^4^ The C-terminal region of wild-type NUP98 contains an RNA-binding domain and an autoproteolytic cleavage site, where the NUP98 precursor protein is cleaved into 90 and 8 kDa peptides.^5^ In addition, it participates in the regulation of gene expression within biomolecular condensates that were termed GLFG bodies.^4,6^ Without exception, all NUP98 fusions join the N-terminus of NUP98 including the FG repeats to the C-terminus of fusion partners, which often contain domains with roles in epigenetic modifications and/or transcriptional control. More than 30 gene partners have been reported for NUP98 in a wider range of AML subtypes including monocytic, megakaryoblastic, and erythroid AMLs, with *NUP98::NSD1* and *NUP98::KDM5A* as the most prevalent.^2,3,7,8^ The resulting fusion proteins promote leukemia by recruiting various chromatin regulatory proteins, leading to the transcriptional activation of leukemia-associated genes.^9–11^

*NUP98*-rearranged (*NUP98*-r) AML is characterized by a distinct gene expression pattern including elevated expression of *HOXA* and *MEIS1* genes, which is similar to that of *NPM1*-mutated (*NPM1*-mut) and *MLL*-r AML, with the exception that *HOXB* genes are also overexpressed in *NUP98*-r and *NPM1*-mut AMLs.^3,12^ Recently, we have also demonstrated that *NUP98*-r AMLs, along with *NPM1*-mut and *MLL*-r AMLs, cluster together in principal component analyses.^13^ In line with this shared transcriptional pattern, NUP98 fusion proteins interact with various chromatin-modifying complexes, including mixed lineage leukemia (MLL) complexes. MLL has been shown to recruit NUP98 fusion proteins to its target genes and is required for the gene expression signature and proliferation of *NUP98*-r AML cells.^14^ The interaction between the N-terminal moiety of MLL and its cofactor Menin is critical in *MLL*-r leukemia, and therapeutic disruption of this association represents a promising targeting strategy that is under current clinical evaluation for this AML type. To date, 3 different Menin inhibitors (SNDX-5613, revumenib; KO0539, ziftomenib; JNJ-75276617) are under clinical investigation with all 3 inhibitors cause dissociation of the Menin–MLL complex. Unlike its 2 sister compounds, revumenib is a CYP3A4 substrate resulting in parallel dose finding studies with and without CYP3A4 inhibitors.^15^ These phase 1 studies have already shown that inhibiting the MLL–Menin interaction with Menin inhibitors reverses leukemic gene expression and induces differentiation of MLL-r leukemic cells, resulting in clinical remission.^15–19^

The physical interaction of NUP98 fusion proteins with MLL complexes at promoters in combination with the similar leukemia-associated gene signature in *NUP98-r*, *NPM1*-mut, and *MLL-r* AML raises the question whether *NUP98*-r AML is dependent on the Menin–MLL interaction. Using cell lines and PDX mouse models expressing *NUP98::HOXA9*, *NUP98::JARID1A*, and *NUP98::NSD1* fusions, Heikamp and colleagues recently demonstrated that interrupting the Menin–MLL interaction disturbs global gene expression and halts leukemic progression in *NUP98*-r AMLs, but no primary samples were studied.^20^

In addition, combined targeting of Menin and secondary lesions such as FLT3-ITDs has not been studied previously in patient-derived *NUP98*-r AML. In this study, applying an ex vivo coculture approach, we found that primary *NUP98*-r AMLs are sensitive to the Menin inhibitor revumenib (SNDX-5613) (EHA abstract 2021). Menin inhibition impaired leukemic proliferation and clonogenicity and induced myeloid differentiation in primary patient-derived AML samples that was associated with global gene expression changes. As the vast majority of *NUP98*-r cases are also *FLT3* mutated,^2,21,22^ we combined Menin and FLT3 inhibition, which caused a strong synergistic suppression of the expansion of patient-derived *NUP98*::*NSD1 FLT3-ITD* double-positive AML cells ex vivo. Importantly, Menin inhibition completely blocked disease progression by eradicating leukemic blasts in a patient-derived NUP98::NSD1/FLT3-ITD-positive PDX model, suggesting that the *NUP98* fusion drives the leukemia. Overall, the results of this study support the clinical evaluation of Menin inhibitors in *NUP98*-r AML patients.

## MATERIALS AND METHODS

### Culture of primary AML cells

Primary AML cells were cultured with healthy bone marrow-derived mesenchymal stem cells (MSCs) under serum-free conditions. MSCs were seeded at a density of 7500 cells/cm^2^ in DMEMlow Glucose plus Glutamax supplemented with 20% fetal bovine serum, 8 ng/mL Fibroblast Growth Factor-beta (FGF2; PeproTech, London, UK) and 100 U/mL penicillin/streptomycin (Gibco BRL, Life Technologies, Breda, The Netherlands), and cultivated at 37°C in 5% CO_2_ overnight or until reaching 50%–70% confluence. Primary AML cells were thawed and added to the MSC layer at a density of 5 × 10^5^ cells/mL in SFEMII medium supplemented with 100 U/mL penicillin/streptomycin, 10 ng/mL IL-3, 10 ng/mL FLT3 ligand, 10 ng/mL granulocyte-macrophage colony-stimulating factor (GM-CSF), 150 ng/mL stem cell factor (SCF), 100 ng/mL thrombopoietin (TPO) (all cytokines were purchased from PeproTech), 750 nM SR1 (Biogeme, Lausanne, Switzerland), and 1.35 µM UM729 (STEMCELL Technologies, Cologne, Germany). Cocultures were incubated at 37°C in 5% CO_2_ and expanded with readjusting AML cell numbers to 5 × 10^5^ cells/mL every 4 days.

### Inhibitor treatment

**Figure 1.**
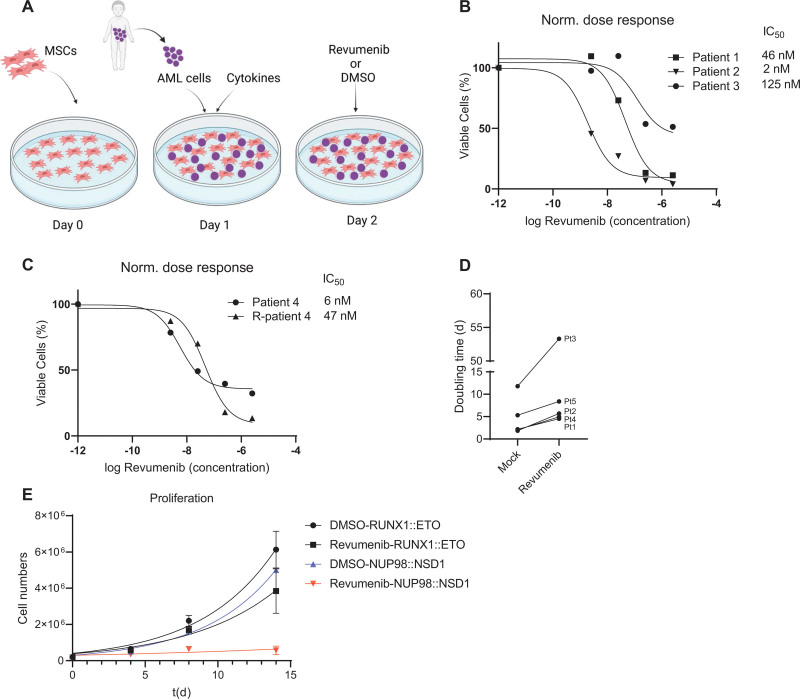
***NUP98*-rearranged (*NUP98-r*) AML cell growth is inhibited upon treatment with revumenib.** (A) Schematic representation of the human bone marrow-derived MSCs coculture platform used to culture primary AML cells. MSCs serve as feeder layers and support AML cell proliferation. Figure created with BioRender.com. (B and C) Normalized dose-response curve of primary *NUP98::NSD1* (*FLT3*-ITD+) AML cells treated with revumenib for 2 weeks. (D) Doubling time of primary AML cells in coculture treated with DMSO or revumenib (250 nM). (E) Proliferation curves of primary AML cells harboring *RUNX1::ETO* and *NUP98::NSD1* under treatment of revumenib (250 nM). Data were presented as mean (±SD). AML = acute myeloid leukemia; MSCs = mesenchymal stem cells.

Primary AML cells were kept in coculture for 4 days, replated on fresh MSCs, and treated with different concentrations of revumenib or 0.1% DMSO (mock). AML cells were replated at a cell concentration of 5 × 10^5^ cells/mL every 4 days on fresh MSCs over a period of 14 days with concomitant inhibitor replenishment (Figure 1A). During this period, untreated leukemic stem cells (CD34+ and CD45RA+) expanded >12-fold with doubling times in the range of 2–3 days. For determining cell viability, the number of viable cells was counted at different time points using 0.4% trypan blue exclusion (Invitrogen, T10282). The ratio of viable cells in DMSO-treated groups versus revumenib-treated groups was plotted to calculate IC_50_ values (Prism 8, GraphPad). The differentiation status of revumenib-treated or DMSO-treated cells was characterized at day 14 by flow cytometry using CD11b-APC-Cy7 (Biolegend, San Diego, CA), by quantitative polymerase chain reaction (qPCR) using *ITGAM* (*CD11b*)-specific and *MNDA*-specific primers and by Wright-Giemsa-stained cytospin slide preparations. For combinational treatment, serial concentrations of revumenib and gilteritinib were used at a constant 4:1 ratio. The highest concentrations were 2.5 µM and 625 nM for revumenib and gilteritinib, respectively. AML cells were treated with revumenib for 10 days followed by the addition of gilteritinib and cell count on day 13.

### In vitro studies and statistical analysis

See the Supplemental Methods for flow cytometry, apoptosis analysis, RNA-sequencing, cytospin analysis, real-time qPCR, clonogenic assay in methylcellulose, western blotting, data quantification, and statistical analysis.

### AML xenograft model

NSG mice were injected intrafemorally with 10^6^ primary AML cells from patient 1 harboring the *NUP98::NSD1* fusion with concomitant *FLT3*-ITD mutation (Suppl. Table S1). Three months after confirmed engraftment, mice were randomized into 3 groups and were fed on a chow diet of 0.01% revumenib, 0.033% revumenib, and normal chow diet. Peripheral blood was collected at different time points and the fraction of CD45+ CD33+ CD34+ human AML cells was assessed via flow cytometry. Mice were humanly killed upon weight loss exceeding 20%, showing clinical signs of leukemia or at the experimental end point.

## RESULTS

### Revumenib inhibits growth of primary patient-derived NUP98-r AML cells

To test the sensitivity of *NUP98*-r AML to the Menin inhibitor, we used transgenic cells ectopically expressing NUP98 fusions and patient-derived primary AML cells containing *NUP98* rearrangements. We explored Menin dependency with revumenib, a Menin inhibitor currently under clinical evaluation.^23^

We first examined the impact of Menin inhibition on cells transduced with *NUP98::NSD1* and *NUP98::DDX10* fusion genes.^24^ Revumenib suppressed cell proliferation of both transduced AML cell lines in a dose-dependent fashion with IC_50_ values of 56 and 36 nM, respectively (Suppl. Figure S1A-S1C).

**Figure 2.**
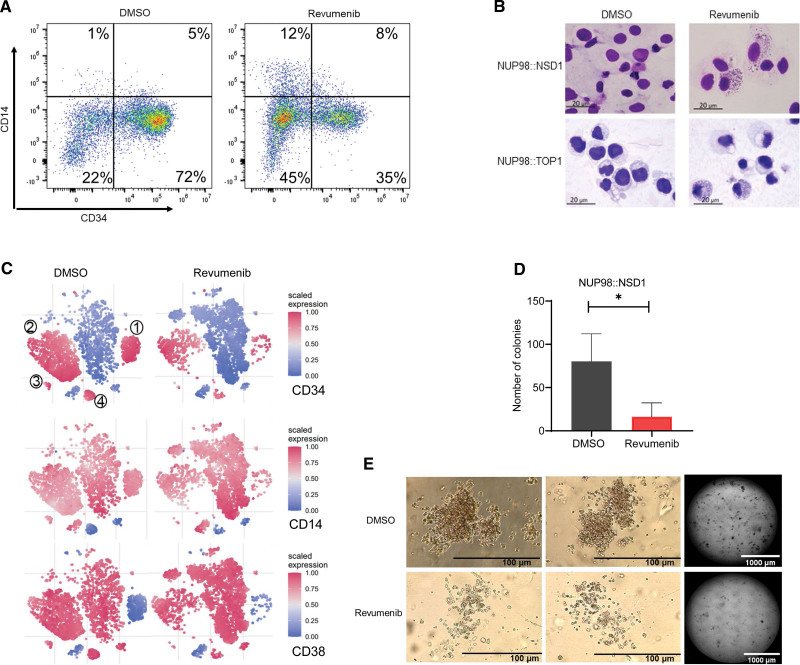
**Menin inhibition induces differentiation of *NUP98*-r primary AML cells.** (A) Representative flow cytometry data showing downregulation of CD34 and upregulation of CD14 expression in primary patient AML cells (*NUP98::NSD1*, *FLT3*-ITD+) upon treatment with revumenib (2500 nM). (B) Wright-Giemsa staining of primary AML cells 2 weeks after exposure to revumenib (250 nM). (C) t-SNE analysis showing distribution of subpopulations in 5 *NUP98::NSD1* primary AML samples based on the expression of 8 cell surface markers. The t-SNE plots depict that immature subpopulations with CD34+ and CD38− markers decrease upon treatment with revumenib. The color is relative to the range of intensities in all cells. (D and E) Clonogenic potential of *NUP98::NSD1* primary AML cells after treatment with revumenib (250 nM) as measured by a methylcellulose-based colony forming assay. Data were presented as mean (±SD). AML = acute myeloid leukemia; t-SNE = t-stochastic neighbor embedding.

Next, we investigated the impact of Menin inhibition on the propagation of patient-derived primary AML cells comprising 5 *NUP98::NSD1* and 1 *NUP98::TOP1* AML sample. The *NUP98::NSD1* samples included a matched sample from the same patient at presentation and relapse (Patient 4). Sequence analysis revealed that all *NUP98::NSD1* samples expressed 2 chimeric fusion mRNAs, with the joining of *NUP98* exon 12 to *NSD1* exon 6 being the predominant transcript. The minor splice variant was the fusion of *NUP98* exon 11 and *NSD1* exon 6 (Suppl. Figure S1E). Importantly, all *NUP98::NSD1* samples carried *FLT3*-ITD at allelic frequencies >0.4, while the *NUP98::TOP1* sample carried a combination of *WT1* and *CEBPA* mutations (Suppl. Table S1). We exposed AML cells to a range of revumenib concentrations between 2.5 and 2500 nM. Coculture on human bone marrow-derived MSCs maintained proliferation of primary AML samples with doubling times ranging from 2 to 10 days (Figure 1D and 1E; Suppl. Figure S1F). Revumenib treatment resulted in a profound dose-dependent suppression of proliferation that was associated with 2-fold increased doubling times and IC_50_ values ranging from 2 to 120 nM (Figure 1B–1E). In contrast, AML cells expressing the RUNX1::ETO fusion protein were unaffected by revumenib at a concentration of 250 nM (Figure 1E; Suppl. Figure S1D).^25^ Revumenib inhibited cell proliferation with a 10-day latency, which is consistent with the inhibitory effect of revumenib on *NUP98::NSD1* and *NUP98::DDX10* transduced cells. This observation is also in agreement with the impact of the Menin inhibitor MI-503 on MLL-rearranged leukemia cells,^26,27^ and other inhibitors of epigenetic factors, for example, DOT1L^28^ and EZH2.^29^ Menin inhibition resulted in a slight increase in apoptotic cells after 2 weeks, which might be a consequence of myeloid differentiation (Suppl. Figure S2A). Instead, combined histology, qPCR, and flow cytometric analysis revealed a dose-dependent decrease of CD34 and CD45RA and a gain of CD11b, CD14, and *MNDA* (Figure 2A and 2B; Suppl. Figure S2B-S2F), indicating that revumenib treatment induced myelo-monocytic differentiation. To investigate more comprehensively how Menin inhibition affects differentiation and the distribution of subpopulations of leukemia cells in NUP98::NSD1 (*FLT3*-ITD+) and NUP98::TOP1 (*WT1*+, *CEBPA*+) primary AML cells, we examined the surface expression of CD14, CD33, CD11b, CD38, CD90, CD34, CD117, and CD45RA. The t-stochastic neighbor embedding analysis identified 4 distinct CD34+ cell clusters (Figure 2C). Revumenib treatment led to a strong reduction of all CD34+ cell clusters 1–4 and a corresponding increase in CD34-cell populations. This increase was associated with changes in the distribution of CD14 and CD38 markers within the cell populations. Examination of the clonogenic potential of NUP98::NSD1-harboring cells as a surrogate marker for malignant self-renewal revealed that revumenib reduced colony numbers >50% as compared with DMSO controls, and the remaining colonies had a more dispersed appearance and were smaller (Figure 2D and 2E). These data demonstrate that treatment with revumenib induces myelo-monocytic differentiation in primary *NUP98*-r cells and suggest that Menin inhibition causes a concomitant loss of self-renewal even in the presence of the *FLT3*-ITD mutation.

### Revumenib induces downregulation of *NUP98* fusion target genes including *FLT3*-ITD

**Figure 3.**
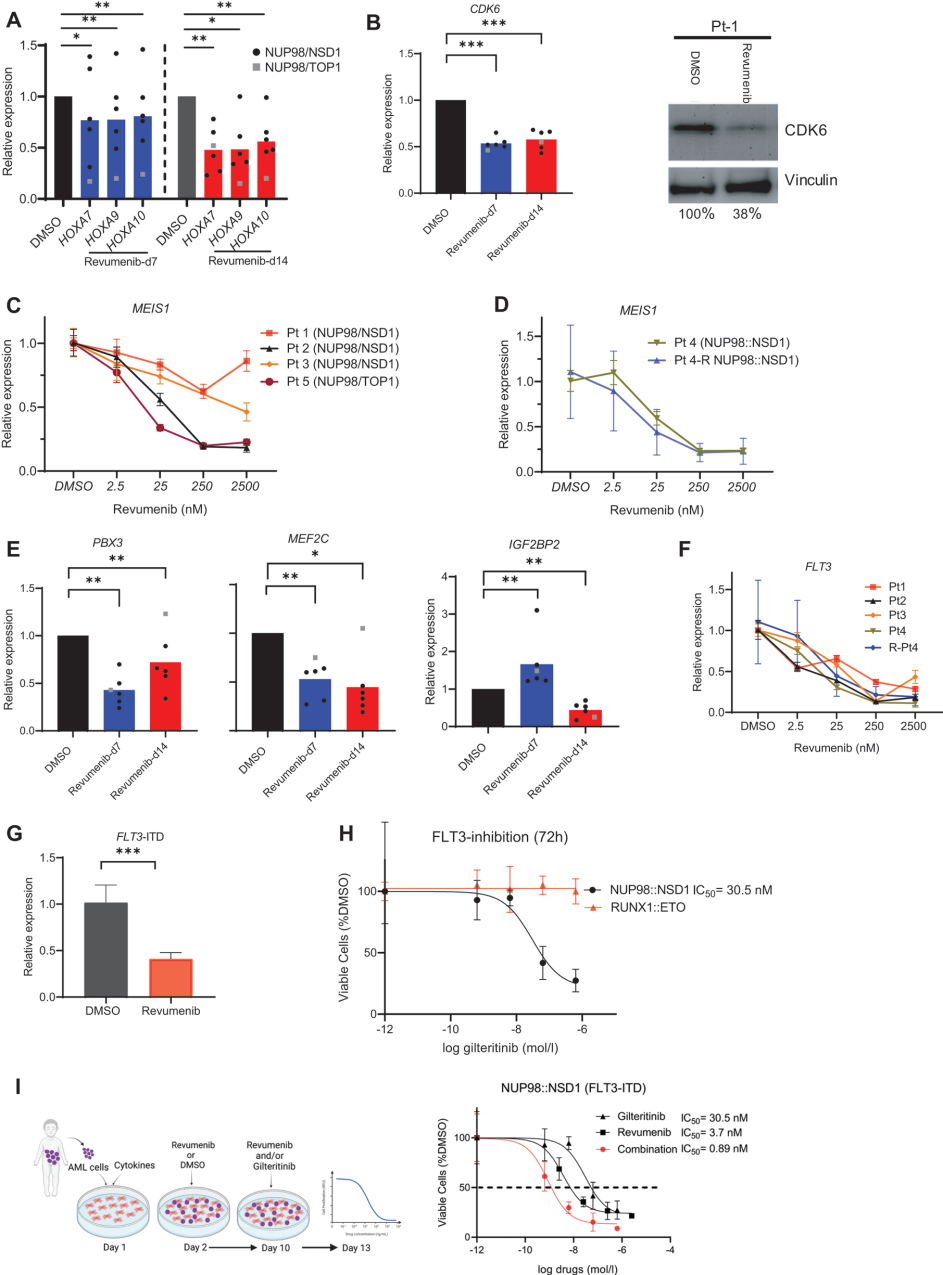
**Menin inhibition suppresses the expression of NUP98 fusion target genes.** Data were presented as mean (±SD). (A) qPCR results showing significant downregulation of NUP98 fusion target genes including *HOXA7-10*, (B) *CDK6*, (E) *MEF2C*, *IGF2BP2*, and *PBX3* in *NUP98*-r primary AML samples upon treatment with revumenib (250 nM). (B) Suppression of CDK6 after 14 days of treatment was confirmed at protein level by western blot. (C, D, and F) qPCR results showing expression of *MEIS1* and *FLT3* after 7 days of treatment. (G) *FLT3*-ITD expression was downregulated after Menin inhibition as measured by qPCR with ITD-specific primers 1 week after treatment. (H and I) Dose-response curves of patient-derived *NUP98::NSD1* (*FLT3*-ITD+) and *RUNX1::ETO* AML cells treated with gilteitinib, revumenib, or their combination. Dashed line represents IC_50_ value. AML = acute myeloid leukemia; qPCR = quantitative PCR.

Like *MLL*-r AMLs, AMLs harboring a *NUP98* rearrangement are characterized by high expression of *MEIS1* and the *HOXA* cluster genes. As the expression of these genes was shown to be dependent on a functional MLL–Menin interaction,^10^ we examined the impact of inhibiting this interaction on the expression of *HOXA7-10* and the genes coding for *MEIS1* and *PBX3* in both *NUP98::NSD1*- and *NUP98::TOP1*-positive primary AML cells. *NUP98::TOP1*-cells showed early downregulation of *HOXA7*, *9,* and *10* genes in the first week (Suppl. Figure S3A), whereas *NUP98::NSD1*-cells showed a significant reduction of *HOXA* gene expression in the second week of inhibitor treatment (Figure 3A), which is consistent with the kinetics of revumenib-mediated inhibition of cell proliferation. In contrast, expression of *CDK6,* a direct target gene of NUP98 fusion proteins, was markedly downregulated already at day 7 of treatment (Figure 3B). Primers are listed in the Supplemental Digital Content (Suppl. Table S4). We observed a dose-dependent downregulation of *MEIS1* in all *NUP98*-r primary samples including a relapse sample (Figure 3C and 3D), while *PBX3, IGF2BP2*, and *MEF2C* expression were variably affected by revumenib (Figure 3E). Interestingly, expression of *FLT3,* a downstream target gene of *MLL* and *MEIS1*, was suppressed in a dose-dependent fashion in all *NUP98-r* samples with a more than 3-fold reduction at 250 nM of revumenib (Figure 3F). These data show that Menin inhibition can target *FLT3* expression in line with similar observation reported for *MLL*-r and *NPM1*-mut AMLs.^16,27^ Because all tested NUP98::NSD1 AML cells were positive for *FLT3-ITD* with allelic frequencies above 0.4, we also evaluated the impact of Menin inhibition on the expression of the mutated allele. We observed a 3-fold reduction of *FLT3*-ITD transcript levels upon exposure to revumenib (Figure 3G), suggesting that therapeutic modulation of the MLL–Menin interaction affects both *NUP98* fusion- and FLT3-ITD-driven leukemic programmes. Nonetheless, the residual *FLT3*-ITD gene expression after treatment with revumenib may persist and be advantageous to leukemic cells. As there are reports indicating that incomplete eradication of this aberration in patients with *FLT3*-ITD+ leukemia may lead to relapse,^30,31^ the moderate *FLT3*-ITD inhibition by revumenib raises the question if concomitant inhibition of the Menin–MLL interaction and FLT3-ITD signaling resulted in an even stronger reduction of leukemic propagation. In support of this hypothesis, it has been previously reported that the combined Menin and FLT3 inhibition exerts synergistic effect in *MLL*-r and *NPM1*-mut leukemias.^27,32^ We therefore examined if the FLT3 inhibitor gilteritinib also synergises with revumenib in interfering with the expansion of *NUP98::NSD1 FLT3*-ITD*+ patient-derived AML cells.* FLT3 inhibition was found to be highly efficient against these AML cells (IC_50_= 30 nM), while primary AML cells (RUNX1::ETO) without *FLT3* mutation were unaffected (Figure 3H). Considering that the Menin inhibitor (revumenib) has shown to suppress *NUP98::NSD1* AML cells after 7–10 days, while FLT3-i induces rapid cytotoxicity, we treated AML cells with Menin inhibitor for 10 days followed by addition of FLT3-i for another 3 days. The combination of Menin and FLT3 inhibitors was highly synergistic with a combination index of 0.27 and strongly suppressed cell proliferation with significantly lower IC_50_ values of both drugs compared with single-drug treatment (Figure 3I). Taken together, although Menin inhibition alone impairs leukemic progression of *NUP98*-r/*FLT3*-ITD+ AML, the combination of Menin and FLT3 inhibitors increases their antileukemic efficacy in a synergistic manner.

### Revumenib induces global gene expression changes in primary patient-derived *NUP98::NSD1* AML cells

**Figure 4.**
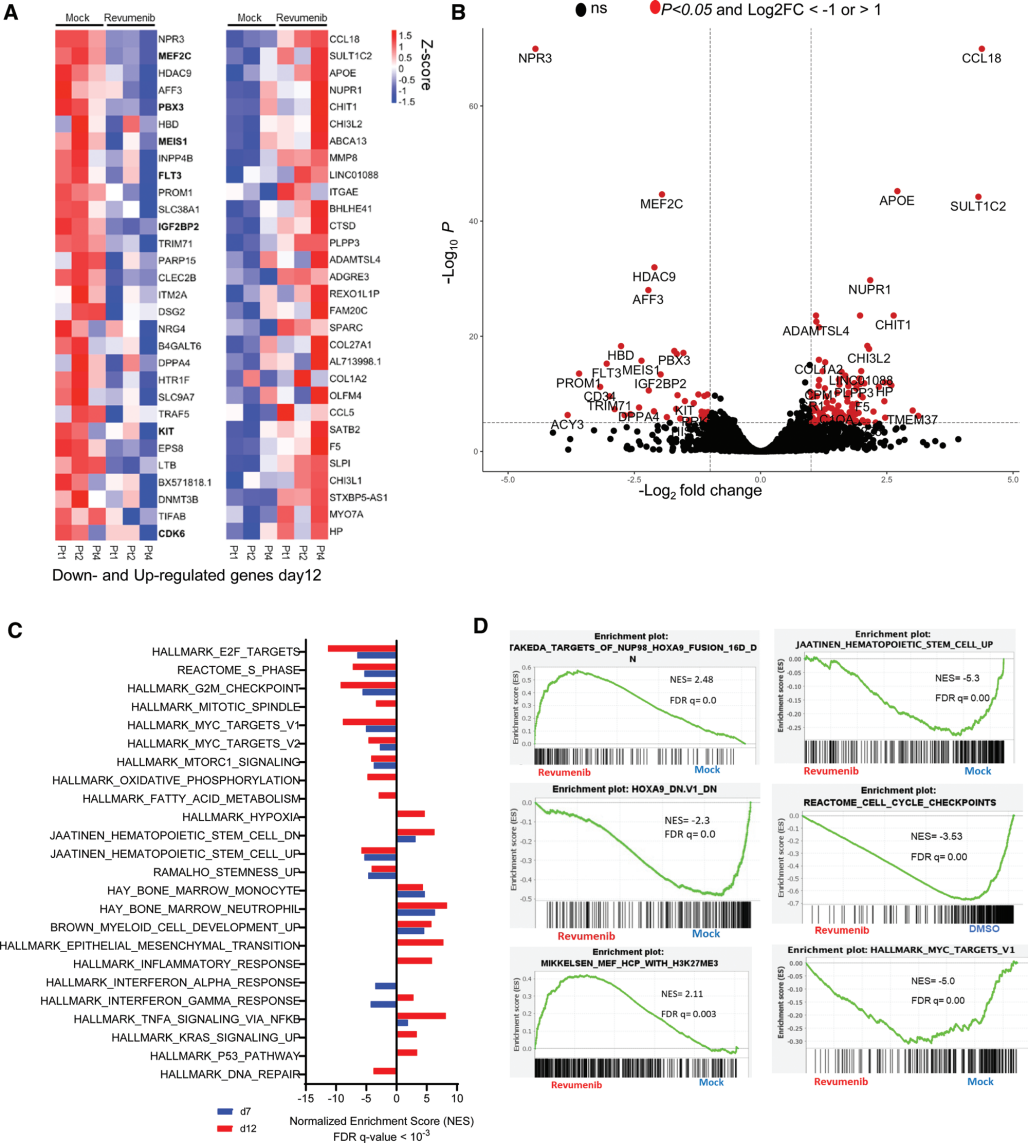
**Gene expression changes of primary AML samples (*NUP98::NSD1*, *FLT3*-ITD+) associated with revumenib treatment.** (A) Heat map of RNA-Seq analysis from 3 different primary patient samples (*NUP98::NSD1*, *FLT3*-ITD), representing top 30 differentially expressed genes 12 days after treatment with revumenib (250 nM). (B) Volcano plot illustrating differentially regulated genes in *NUP98::NSD1* patients (n = 3) treated with revumenib (250 nM) compared with DMSO-treated samples (n = 3) on day 12 of treatment. Each dot represents a transcript with detectable expression. The FC (in log 2 scale) of the transcripts is plotted on the x-axis and the statistical significance expressed as −log10(FDR) on the y-axis. Genes not classified as differentially expressed are shown in black. (C and D) GSEA analysis of RNA-seq data showing enriched gene sets and pathways in revumenib (250 nM)-treated samples compared with DMSO-treated groups. AML = acute myeloid leukemia.

To characterize global gene expression changes in response to the inhibition of the MLL–Menin interaction, we performed RNA-sequencing with sorted AML cells from 3 patients after 7 and 12 days of exposure to revumenib or vehicle. Treatment with revumenib led to profound changes in gene expression pattern with 150 and 183 significantly (≥2-fold change, padj < 0.05) downregulated and upregulated genes, respectively (Suppl. Table S2). Extending the treatment time to 12 days increased the number of upregulated genes to 422 (Figure 4A and 4B; Suppl. Table S3). Genes downregulated at both time points include genes encoding key hematopoietic transcription factors (eg, *MEIS1, PBX3*, and *MEF2C*), receptor tyrosine kinases (*FLT3* and *KIT*) and factors associated with stemness such as *CD34*, *DNMT3B*, and *PROM1*. Upregulated genes include members of the AP-1 transcription factor family (*FOS, FOSB,* and *JUNB*), inflammatory molecules (*SGK1*, *CXCL2*, and *IL1R1*) and factors involved in myeloid differentiation (eg, *FGR, MMP8*, and *S100A8*).

Gene set enrichment analysis revealed a strong inverse correlation between the gene expression signature induced by Menin inhibition and the NUP98::HOXA9 fusion protein signature, further supporting the notion that interference with the MLL–Menin interaction directly affects the core transcriptional programme of NUP98 fusion proteins. Furthermore, there was a highly significant overlap of genes downregulated by menin inhibition between our study and Heikamp and colleagues^20^ (Suppl. Figure S4A and Suppl. Table S5). Moreover, the MLL–Menin inhibition was associated with loss of *MYC*-target genes, impaired cell cycle progression, loss of leukemic stemness, and higher H3K27me3 levels indicating increased EZH/PRC2 activity (Figure 4C and 4D). Overall, these data demonstrate that inhibition of the Menin–MLL interaction blocks leukemic self-renewal potential and reactivates myeloid differentiation in *NUP98*::*NSD1* primary AML cells.

### Revumenib eradicates leukemic cells in a PDX model of AML with *NUP98::NSD1* (*FLT3*-ITD)

**Figure 5.**
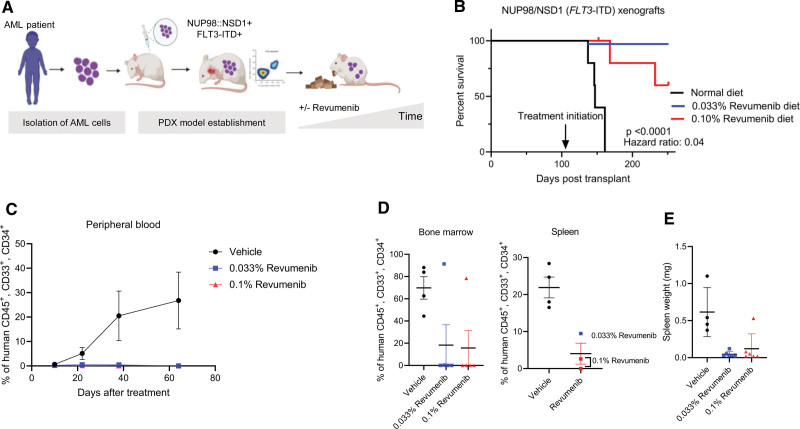
**In vivo efficacy of revumenib in a *NUP98::NSD1* (*FLT3*-ITD) AML PDX model.** (A) Schematic representation of the in vivo study. (B) Kaplan–Meier survival plots showing the median survival upon treatment with revumenib or vehicle. Arrow indicates begin of treatment. (C) Flow cytometric quantification of human CD45+ cells in peripheral blood of *NUP98::NSD1 FLT3*-ITD PDX mice during treatment with revumenib or vehicle. Mean ± SE, n = 5 per group. (D) Flow cytometry data showing the frequency of leukemic blasts (hCD45+, hCD34+, hCD33+) in the spleen and bone marrow of *NUP98::NSD1* (*FLT3*-ITD) PDX mice. (E) Spleen weight of *NUP98::NSD1* (*FLT3*-ITD) PDX mice. AML = acute myeloid leukemia.

Next, we examined the dependency of *NUP98*-r AML engraftment and in vivo propagation on a functional MLL–Menin interaction. We therefore tested the efficacy of revumenib in a *NUP98::NSD1* PDX mouse model (Figure 5A). NSG mice were intrafemorally transplanted with *NUP98::NSD1*+ *FLT3*-ITD+ PDX cells derived from patient 1. Revumenib treatment was initiated after confirming engraftment by continuous provision of chow containing 0.033% or 0.1% revumenib. Control animals showed progressing disease as indicated by increasing blast counts, whereas revumenib-treated animals experienced no increase in the number of peripheral blood leukemic cells over the whole observation period (Figure 5C). While the median survival of control animals was 147 days, all animals of the low dose revumenib group reached the end point of the study without showing clinical signs of disease. In the high dose group, 2 animals died without detectable leukemia, and 2 with pale bone marrow and splenomegaly (Figure 5B–5E). Postmortem inspection at the end of the study identified 1 additional animal from the low dose group with leukemic infiltration of the bone marrow and the spleen. Importantly, revumenib treatment completely eradicated leukemic blasts (hCD45+, hCD34+, and hCD33+) in the remaining 8 mice (Figures 5D and 5E). Taken together, these data show that revumenib has potent antileukemic activity toward *NUP98::NSD1* AML even with *FLT3*-ITD as cooccurring mutation.

## DISCUSSION

Here, we show that the phenotype and leukemic signature of *NUP98*-r AML cells are dependent on a functional MLL–Menin interaction in primary AML samples. Blocking this interaction with a Menin inhibitor interfered with leukemic transcriptional programmes associated with impaired proliferation, clonogenicity and facilitated myeloid differentiation, and prevented engraftment of *NUP98::NSD1 FLT3*-ITD patient cells in immunodeficient mice.

Previous studies addressing the effect of Menin inhibition in AML have used genetically engineered cell lines or characterized effects of Menin inhibition on *NPM1*-mutated, *MLL*-r or with *NUP98*-r mouse PDX models, demonstrating that the MLL–Menin interaction is a dependency in these AML types.^16,20,26,27,33^ We have now extended these investigations by examining the impact of MLL–Menin inhibition in primary patient AML cells expression NUP98 fusion proteins.^20,33–35^ Transgenic cell lines have been instrumental in investigating core mechanisms by which NUP98 fusion proteins drive leukemia.^14,20,24,36,37^ However, they do not reflect the evolutionary processes of leukemogenesis and the clonal heterogeneities linked to this process. Furthermore, the interplay of the major driver oncogene with secondary mutations that are present in patients and the interactions of AML cells with its niche cannot be adequately studied using these models, although these aspects are highly relevant for assessing drug response. Our ex vivo results extend previous findings on the effect of inhibiting MLL–Menin interaction in *NUP98*-r AML. The ex vivo evaluation of epigenetic inhibitors such as Menin inhibitors require culture conditions that preserve the differentiation status of different AML subpopulations for prolonged periods. Therefore, we examined the ex vivo effects of revumenib by using a coculture system that maintains and expands patient-derived primary AML cells for >2 weeks. Using this platform, we could demonstrate that revumenib induced myeloid differentiation and suppressed proliferation in 5 different primary *NUP98::NSD1* AML samples but does not trigger apoptosis. Moreover, we also showed that this treatment resulted in marked changes in *NUP98::NSD1*-driven transcriptional networks that also included the downregulation of *FLT3*-ITD, which is a very common mutation in *NUP98*-r AML. *FLT3* has previously been shown to be a direct transcriptional target of MEIS1/HOXA9, suggesting that inhibition of the MLL–Menin interaction targets *FLT3* and possibly *FLT3*-ITD expression via downregulation of MEIS1/HOXA.^27,38–40^

In AMLs where FLT3-activating mutations, and in particular *FLT3*-ITD, are present, FLT3 is a critical therapeutic target.^41,42^ There are currently several FLT3 inhibitors in clinical use but achieving to long-term remission when it is used as a single agent is challenging.^27,42,43^ *FLT3*-ITD is a secondary genetic event in in our patient-derived *NUP98*-r AML and its signaling may also cooperate via STAT5 with MLL–Menin complexes in regulating gene expression.^27^ Here we showed that Menin inhibition suppresses *FLT3*-ITD expression and also increases this AML subtype susceptibility to FLT3 inhibitors. Additionally, we demonstrate that Menin inhibition single treatment is sufficient for suppressing *NUP98*-r AML in the presence of concomitant mutations such as *FLT3*-ITD and *WT1*; however, the combination of Menin and FLT3 inhibitors significantly reduce the antileukemic efficacy of each agent. Furthermore, these findings highlight the therapeutic interference with the MLL–Menin interaction as a paradigm for affecting both the primary and a secondary genetic event at the functional (NUP98 fusion protein) and at the transcriptional (*FLT3*-ITD) level. In a clinically relevant *NUP98::NSD1* PDX model, we found that MLL–Menin inhibition can completely eradicate this aggressive AML subtype even when a concurrent *FLT3*-ITD mutation was present.

Using transduced cells, Thanasopoulou et al previously reported that the expansion of *NUP98::NSD1/FLT3*-ITD+ AML cells is accelerated by FLT3-derived signals. Our findings demonstrate that expression levels of *FLT3*-ITD and also cell proliferation decrease upon ex vivo revumenib treatment of patient-derived *NUP98::NSD1*/*FLT3*-ITD+ AML cells suggesting that the NUP98 fusion is the main driver of leukemia development.^22^

In this study, we show that the *NUP98*-r AMLs with different fusion partners such as NSD1, TOP1, and DDX10 are sensitive to Menin inhibition. However, considering the diversity of fusion partners, which fall into distinct leukemia phenotypes such as AMKL (NUP98::JARID1A) and the variety of epigenetic regulatory domains in this AML type, more samples are required to be tested to determine if all NUP98-r AMLs are dependent on the MLL–Menin interaction. For example, AMLs expressing NUP98::KMT2A have been reported to not react to Menin inhibition.^44^ This AML subtype, unlike other *NUP98*-r AMLs, does not show elevated expression of *HOX* genes and therefore might follow alternative mechanism of leukemogenesis. This could be attributed to the absence of exon 1 in *KMT2A* within *NUP98::KMT2A* fusion, which encodes the Menin interaction domain. Further research is needed to determine whether wild-type KMT2A in this AML subtype has any defining role in sensitivity to Menin inhibition.

Heikamp et al recently demonstrated the responsiveness of the *NUP98::NSD1* PDX mouse model, which was also *WT1* mutant, to Menin inhibition. However, the study identified another PDX model with additional mutations (*ASXL1*, *IDH1*, *BCORL1*, *WT1*, and *FLT3*-ITD) that did not respond to Menin inhibition, indicating that certain mutations or their combinations may confer resistance to Menin inhibition.^20^ *FLT3*-ITD and *WT1* are the most prevalent concurrent secondary genetic alterations in this AML subtype, and their cooccurrence with *NUP98* fusions confers a poor prognosis.^2,3,21,22,45,46^

Our ex vivo and in vivo results with *NUP98*-r AML models suggest that samples expressing mutated *WT1* or *FLT3*-ITD respond favorably to MLL–Menin inhibition, indicating that these mutations do not necessarily lead to treatment resistance. However, with respect to the *BCORL1* mutation, recent studies have suggested that it dynamically competes with NUP98 fusions for transcriptional regulation of developmentally regulated, stem cell-associated genes as a component of the polycomb repressive complex (PRC) 1.1.^47^ Nonetheless, *BCORL1* mutations are uncommon in *NUP98*-r AML and are poorly understood.

Overexpression of *MEIS1/PBX3* is sufficient for transformation of mouse hematopoietic stem cells and driving AML in vivo, as previously reported.^48^ Moreover, elevated expression of *HOXA9* and *MEIS1* genes is associated with a number of hematological malignancies, suggesting that these genes are involved in the development of a variety of leukemias.^49–51^ Patients with high *MEIS1* expression have shorter survival times than those with low *MEIS1*, indicating that *MEIS1* expression could be a prognostic factor even in AMLs with normal karyotype.^50,52^ In this context, it is interesting to note that Menin inhibition has profound antileukemic effects in *MLL*-r, *NPM1*-mutant, and *NUP98*-r acute leukemias where *MEIS1/HOXA9* is highly expressed. In contrast, AML cells expressing the RUNX1::ETO fusion protein do not execute a HOXA9/MEIS1 transcriptional programme and are not affected by Menin inhibitors, strongly suggesting a correlation between *HOXA/MEIS1* expression and MLL–Menin dependence. While *HOXA9* expression is moderately suppressed by Menin inhibition, *MEIS1* is one of the first genes to be downregulated upon inhibition of the MLL–Menin interaction. Consequently, high *MEIS1* overexpression may indicate susceptibility of a given AML toward Menin inhibition. Alternatively, comparative analyses of global gene expression patterns and coclustering with *MLL*-r, *NPM1*-mut, or *NUP98*-r AMLs may identify patients potentially benefitting from Menin inhibitors. Future evaluation of a wider range of AML samples will be required to examine these hypotheses.

Taken together, these data show that *NUP98*-r AMLs are highly susceptible toward Menin inhibition even in the presence of cooccurring mutations including *FLT3*-ITD and *WT1*. Consequently, they strongly support the inclusion of patients suffering of this AML subtype in the clinical evaluation of Menin inhibitors.

## AUTHOR CONTRIBUTIONS

MR wrote and edited the article, performed the experiments, interpreted, and analyzed data. HB and ST performed the experiments and analyzed data. KS discussed the study concept and edited the article. RC performed the experiments. MA analyzed data. AKH performed the experiments. FG conceptualized the study, discussed the study concepts, and edited the article. GMM interpreted data. CMZ conceptualized the study, discussed the study concepts, and edited the article. OH designed, conceptualized, and supervised the study, discussed the study concepts, interpreted data, and edited the article.

## DATA AVAILABILITY

Data sharing requests should be sent to Olaf Heidenreich.

## DISCLOSURES

OH received research funding from Syndax. GMM is employee and shareholder of Syndax Pharmaceuticals. All the other authors have no conflicts of interest to disclose.

## SOURCES OF FUNDING

This work was supported by a KiKa programme grant (329) to OH and grants from the Austrian Science Fund (FWF, grants no. TAI-490 and P35628) to FG. ST is the recipient of a DOC fellowship of the Austrian Academy of Sciences at the University of Veterinary Medicine.

## Supplementary Material


