# Supplementary material for: The MLL–Menin Interaction is a Therapeutic Vulnerability in NUP98-rearranged AML
Source: Hemasphere. 2023 Jul 27;7(8):e935. doi: 10.1097/HS9.0000000000000935 (PMC10378738; doi:10.1097/HS9.0000000000000935)
Supplement: Supplementary file 1 [file hs9-7-e935-s001.pdf]

Supplemental

The MLL – Menin interaction is a therapeutic vulnerability in *NUP98*-rearranged AML

Supplemental material and methods:

Primary AML cells

Primary AML samples were obtained from the Princess Maxima Center for pediatric Oncology after written informed consent. Four samples were derived from pediatric patients with de novo and one sample from relapsed AML, all of them contained NUP98 fusions. Four samples were positive for *NUP98::NSD1* with concomitant *FLT3*-ITD mutations and one sample was positive for *NUP98::TOP1* with additional *WT1* and *CEBPA* mutations.

Flow cytometry

For flow cytometric analysis, primary human AML cells were washed and suspended in PBS with 1% FBS. To determine viability, cells were incubated with viakrome 808 (Beckman coulter) for 20 min at 4°C followed by washing and staining with an antibody cocktail containing CD33-PE, CD34-APC, CD14-FITC, CD38-PerCP-Cy5.5, CD11b-APC-Cy7, CD45RA-BV785, CD90-BV421, CD117-BV605 (Biolegend®, San Diego, CA, USA). All flow cytometry experiments were performed on a Beckman Coulter CytoFLEX LX with subsequent data analysis using FlowJo software (V10.0.7, TreeStar, Ashland, OR, USA).

Apoptosis analysis

For the flow cytometric assessment of apoptosis, cells were washed with PBS and resuspended in Annexin V Binding Buffer (10 mM HEPES, 140 mM NaCl, 2.5 mM CaCl2) followed by incubation with fluorescein isothicyanate (FITC, 1:25, Biolegend®, San Diego, CA, USA)-conjugated annexin V and SYTOX™ Deep Red Nucleic Acid Stain (Thermo Fisher Scientific, 2 uM) for 15 minutes at 37 °C. The number of

apoptotic cells, defined as the Annexin V-positive cells, was determined by flow cytometry.

Cell viability assay

Cells transduced with NUP98::NSD1 and NUP98::DDX10 fusion genes were seeded in biological triplicates and treated with revumenib (Syndax Pharmaceuticals) or DMSO, at indicated concentrations every 48 or 72 hours. Cell numbers were determined in regular intervals with the Intellicyt iQue Screener (Sartorius AG, Goettingen, Germany) and analyzed with iQue ForeCyte Software. The data was plotted with GraphPad Prism version 8.0.0 (GraphPad Software, San Diego, California USA).

Cytospin Analysis

To assess cell morphology, cells were washed with PBS and cytocentrifuged onto glass slides. Slides were fixed in methanol for 2 min and stained using Wright-Giemsa staining or with Epredia™ Shandon™ Kwik-Diff™ staining kit (Thermo Fisher Scientific). Slides were imaged on a Leica DM6 (Leica Microsystems) bright-field microscope.

Real-time quantitative PCR

RNA was isolated using the NucleoSpin RNA purification kit (Macherey-Nagel, Düren, Germany) according to the manufacturer's protocol. Concentration and quality of the RNA was determined with a NanoDrop 1000 (ThermoFisher Scientific). For cDNA synthesis, 500 ng of RNA were reverse transcribed using the RevertAid First Strand cDNA Synthesis Kit (ThermoFisher Scientific) according to the manufacturer's protocol. Quantitative real-time polymerase chain reactions (Q-RT-PCR) were performed in triplicates with 1 µL cDNA using SYBR Green gene expression master

mix on a CFX384 Real-Time PCR detection system (Bio-Rad Laboratories, California), 30 seconds at 95°C followed by 35 cycles at 95°C for 15 seconds and 60°C for 30 seconds. Fold change relative to the control condition was calculated by the comparative ΔΔ cycle threshold method using TBP as the housekeeping gene for normalization.

Clonogenic assay in methylcellulose

Primary patient samples were treated with 250 nM revumenib and grown in SFEMII media supplemented with cytokines as described above for 12 days. Cells were washed with PBS and plated in triplicates on methylcellulose media supplemented with human cytokines (Methocult H4435, StemCell Technologies) at a density of 105 cells. Cells were incubated at 37 °C, 5% CO2 for 14 days before colonies were counted. Colonies were quantified using a Leica DMi8 inverted microscope (Leica Microsystems).

Sanger sequencing

To confirm the presence of the *NUP98::NSD1* fusion transcript and activating *FLT3*-ITD mutations in samples Sanger sequencing was used. Briefly, total RNA (500 ng) was extracted from patient samples using the NucleoSpin RNA purification kit (Macherey-Nagel, Düren, Germany) and reverse transcribed to cDNA using RevertAid First Strand cDNA Synthesis Kit (Thermo Fisher Scientific). The *NUP98::NSD1* fusion transcript was amplified from the cDNA using specific primers (Table S4). Purified PCR products were cloned into vectors using pGEM®-T Easy Vector Systems and transformed into Escherichia coli JM109 competent cells according to the manufacturer's protocol (Promega). After overnight incubation at 37°C, *NUP98::NSD1*+ and *FLT3*-ITD+ colonies were identified on LB + ampicillin (100 µg/ml)

agar plates using colony-PCR, followed by selection and cultivation of positive colonies overnight at 37°C. The next day, plasmids were extracted using the QIAprep Spin Miniprep Kit (QIAgen) and sequenced. Sequencing and RT-qPCR demonstrated the presence of two alternative fusion junctions of *NUP98::NSD1* in patient AML samples. The splice variant which joins *NUP98* exon 11 to *NSD1* exon 6 was the predominant splice form. The juxtaposition of exon 12 of *NUP98* with exon 6 of *NSD1* was the minor splice variant. Reciprocal *NSD1/NUP98* expression was also observed, in which exon 6 of *NSD1* is fused to exon 13 of *NUP98*. *FLT3*-ITD was detected in all AML samples with varying ITD lengths.

Colony PCR cycle conditions: 1 cycle of 95°C for 10 min followed by 40 cycles of 95°C for 15 seconds and 60°C for 30 seconds.

Primers are described in the supplementary table S4.

RNA sequencing:

For the in vitro RNA-seq experiment, we treated 3 different NUP98::NSD1 (FLT3-ITD) primary AML samples in 3 independent experiments either with revumenib (250 nM) or DMSO (0.1%). We used FACS to separate leukemic cells from non-leukemic cells, MSCs and debris from co-culture. RNA was isolated from CD45+, CD33+ FACS sorted AML cells using the NucleoSpin RNA purification kit (Macherey-Nagel, Düren, Germany) according to the manufacturer's protocol. For gene expression analysis, reads were mapped to the human genome (Hg38) using STAR (v2.2.0c). Only reads that mapped to unique genomic locations (MAPQ > 10) were used for downstream analysis and differentially expressed genes between the treated groups were identified using DESeq version 2.

Western blotting

Equal numbers of pelleted cells were washed with ice-cold phosphate-buffered saline (PBS), lysed, and protein was extracted using RIPA buffer. Protein concentration was measured using the Pierce BCA Protein Assay. Samples were separated on 8% sodium dodecyl sulfate-polyacrylamide gels and proteins were transferred to nitrocellulose membranes at 120 V for 60 min in 1× transfer buffer. Membranes were blocked with 5% non-fat dry milk in TBS-T for 1 hr at room temperature and incubated with primary antibodies in TBS-T overnight at 4°C. Afterwards, the blots were washed and incubated with HRP-conjugated goat anti-mouse IgG at RT for 1 h. After washing three times for 5 min, protein bands were visualized through chemiluminescence reaction (SuperSignal West Pico, Thermo Scientific) according to the manufacturer's protocol. The intensity of the signals was measured by ImageJ software (version no. 1.8.0; National Institutes of Health, Bethesda, MD, USA).

Data quantification and statistical analysis

RNA-seq and t-SNE analysis were performed as described in the Supplemental Methods. The software GSEA 4.1.0 was used for enrichment analysis of altered biological pathways. Statistical analyses, including non-linear regression analyses, t-tests, and ANOVA were performed with the Prism software package, version 8.0. Data obtained from multiple experiments were reported as mean ± SD. If not stated elsewhere, differences with $p < 0.05$ were recognized as statistically significant.

Supplemental tables

Supplemental Table 1

Individual characteristics of the patient samples used in this study.

ID no.	Fusion	Age, y	Blast%	Sex	Mutations	Relapse/Diagnostic	Karyotyping
Patient 1	NUP98::NSD1	13	96	M	FLT3-ITD	Diagnostic	46,XY[20].nuc
Patient 2	NUP98::NSD1	15	85	F	FLT3-ITD	Diagnostic	47,XX,+6[9]/46,XX[1].nuc ish(MECOMx2)[100],(NUP98x2)(5’NUP98 sep3’NUP98x1)[92/100],(KMT2Ax2)[100],(ETV6x2)[100].arr(6)x3[0.9]
Patient 3	NUP98::NSD1	11	91	F	FLT3-ITD	Diagnostic	NA,
Patient 4	NUP98::NSD1	2.3	92	M	FLT3-ITD	Diagnostic	NK, FAB: M4
Relapse-patient 4	NUP98::NSD1	3	92	M	FLT3-ITD	Relapse	NK, FAB, M4
Patient 5	NUP98::TOP1	13	NA	F	WT1, CEBPA	Diagnostic	NA, FAB: M4

F indicates, female; M, male; NA, not available; FAB, French-American-British subtype; WT1, Wilms tumor1; FLT3-ITD, Internal tandem duplication (ITD) mutation in Fms-like tyrosine kinase.

Supplemental Table 4

list of primers used for qPCR and colony PCR.

Primers (qPCR)	Forward Sequence (5'->3')	Reverse Sequence (5'->3')	Annealing temperature(°C)
HOXA7	CAAAATGCCGAGCCGACTT	TAGCCGGACGCAAAGGG	60
HOXA9	GCTTGTGGTTCTCCTCCAGT	GTTGGCTGCTGGGTTATTGG	60
HOXA10	TCACGGCAAAGAGTGGTC	AGTTTCATCCTGCGGTTCTG	60
Meis1	ACAGCAGTGAGCAAGGTGAT	TTGGGAAAGATGCCACGCTT	60
FLT3 (1)	GCAATTTAGGTATGAAAGCCAGC	CTTTCAGCATTTTGACGGCAACC	60
FLT3 (2)	CACCAGGCTGTTCACAATAGA	CATGAACAGCTTTGCACCTTATC	60
FLT3-ITD	TGGTAAGAATGGAATGTGCCAA	CCCTGCAAAGACAAATGGTGA	60
CDK6	CGGAGAACACCCTTGGTGG	TAGGCGGTTTCCTTGGAGAAG	60
CD11b	TAACATCACCAACGGAGCCC	TTTCTCACTGCGGAAGGCAT	60
MNDA	CATCGGAAGCAAGAGGGAGG	ATGAGGTCTGGGGTAGTGGG	60
HOXB3	TATGGCCTCAACCACCTTTC	AAGCCTGGGTACCACCTTCT	60
HOXB4	CTGGATGCGCAAAGTTCACGTG	CGTGTCAGGTAGCGGTTGTAGT	60
HOXB5	CGAGGGGCAGACTCCGCAAA	GCAGAGTGCGTGGGCGATCT	60
NUP98::NSD1 (1)	TCTTGGAACTGGGCTTGGTG	TTCTTCTCTGACCGCACAGC	66.2
NUP98::NSD1 (2)	TGGACAGGCATCTTTGTT	ACAGCGGGAACTTACCTT	60
NSD1/NUP98	AGTTTGACACCACAGGCTGA	CTGGGCTGCTGGATTTGTTG	60

Supplemental figures:

Supplemental Figure 1

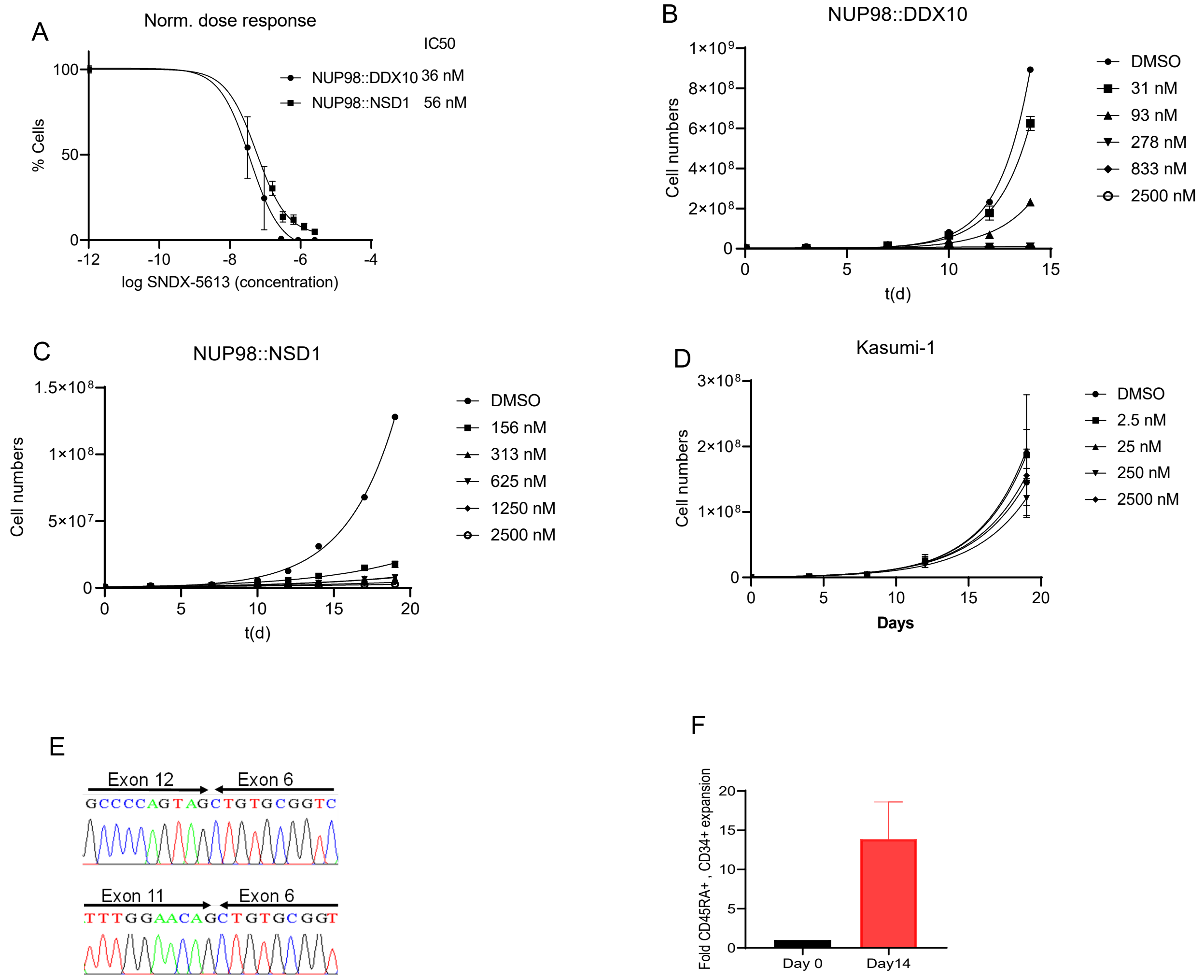

Figure S1: Revumenib has target activity against NUP98 fusions. A) Normalized dose-response curve of NUP98::DDX10 and NUP98::NSD1-transduced cells after treatment with revumenib. B-D) Proliferation curves of transduced NUP98::NSD1, NUP98::DDX10 AML cells and Kasumi-1 cell line upon treatment with DMSO and revumenib. E) Sequencing analysis of PCR product, showing breakpoints of two splice transcripts causing in-frame fusions of NUP98::NSD1 in primary AML cells. The exons involved in the fusions are indicated. F) Bar graph showing expansion of primary AML cells in co-culture. In 2 weeks the viable CD34+, CD45RA+ cell population (patient 2) expanded more than 12-fold.

Supplemental Figure 2

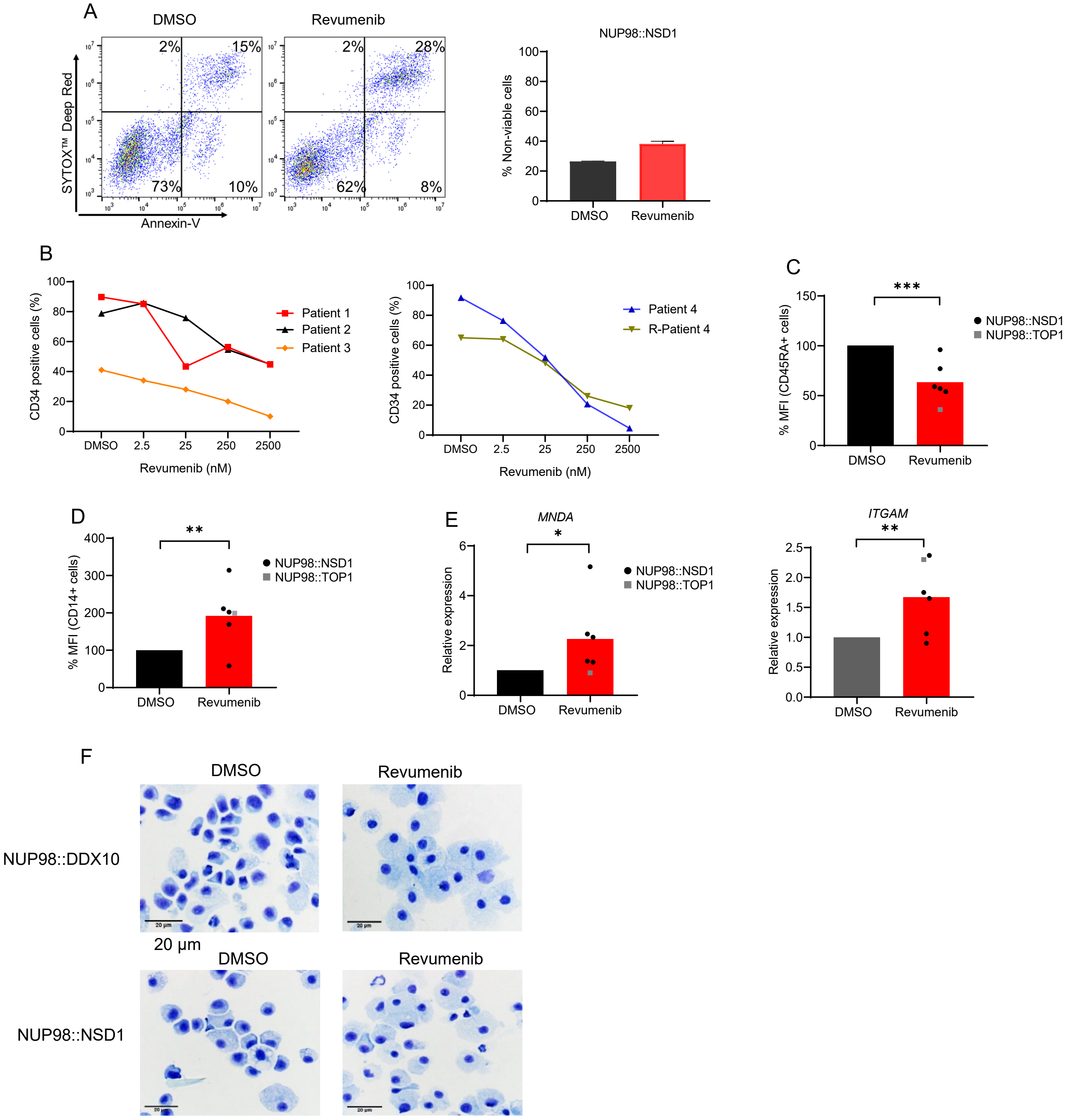

Figure S2: Menin inhibition induces differentiation of NUP98-r AML cells. A) Representative flow cytometry data showing apoptosis of primary AML cells (NUP98::NSD1, FLT3-ITD+) upon treatment with revumenib (250nM) after 2 weeks. B) Flow cytometry results showing dose-dependent suppression of CD34 in all evaluated NUP98::NSD1 diagnostic and relapse primary AML samples. C-E) Quantitative PCR (qPCR) of ITGAM and MNDA mRNA expression, and flow cytometry results (mean fluorescence intensity, MFI) of CD11b, CD14, and CD45RA in NUP98::NSD1 and NUP98::TOP1 cells 2 weeks after treatment with revumenib (250 nM). MFI values were normalized to those of controls (DMSO). F) Wright-Giemsa staining of NUP98::DDX10 and NUP98::NSD1 fusion transduced AML cells after exposure to revumenib (278 and 2500 nM respectively).

Supplemental Figure 3

A

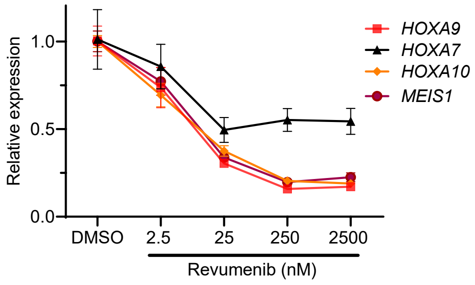

Figure S3: Menin inhibition suppresses the expression of NUP98 fusion target genes. A) Quantitative PCR (qPCR) results showing downregulation of HOXA7-10 and *MEIS1* genes in a dose-dependent manner in NUP98::TOP1 primary AML cells after 7 days of treatment with revumenib.

Supplemental Figure 4

A

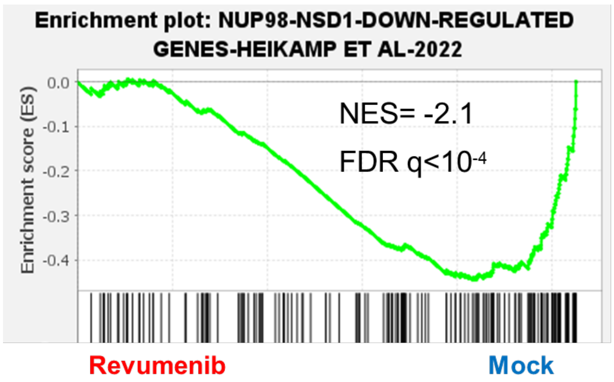

Figure S4: GSEA analysis of RNA-seq data against set of down-regulated genes in a murine NUP98::NSD1 PDX model under Menin inhibition [1] .

1. Heikamp EB, Henrich JA, Perner F, et al. The Menin-MLL1 interaction is a molecular dependency in NUP98-rearranged AML. *Blood, The Journal of the American Society of Hematology*. 2022;139(6):894-906.
